# SPRY4-AS1, A Novel Enhancer RNA, Is a Potential Novel Prognostic Biomarker and Therapeutic Target for Hepatocellular Carcinoma

**DOI:** 10.3389/fonc.2021.765484

**Published:** 2021-10-04

**Authors:** Mu Ye, Sheng Wang, Jing-bo Qie, Pei-long Sun

**Affiliations:** ^1^ Center for Tumor Diagnosis and Therapy, Jinshan Hospital, Fudan University, Shanghai, China; ^2^ Department of General Surgery, Jinshan Hospital, Fudan University, Shanghai, China; ^3^ Institutes of Biomedical Sciences, Fudan University, Shanghai, China

**Keywords:** enhancer RNA, hepatocellular carcinoma, biomarker, therapeutic target, SPRY4-AS1

## Abstract

A growing number of evidence have demonstrated the involvement of enhancer RNAs (eRNAs) in tumor progression. However, the possible functions of eRNAs in hepatocellular carcinoma (HCC) remain largely unclear. Our present research aimed to screen critical eRNAs and to further delve into the clinical significance of eRNAs in HCC patients. In this study, we identified 124 prognosis-related eRNAs by analyzing The Cancer Genome Atlas (TCGA) datasets. Among them, SPRY4 antisense RNA 1 (SPRY4-AS1) may be a key eRNA involved in HCC progression. SPRY4 was a regulatory target of SPRY4-AS1. High SPRY4-AS1 expression was associated with poor prognosis of HCC patients. Kyoto Encyclopedia of Genes and Genomes (KEGG) assays revealed that the mainly enriched biological process included Human papillomavirus infection, Hippo signaling pathway, and Proteoglycans in cancer. Besides, RT-PCR and immunohistochemical staining confirmed SPRY4-AS1 as an overexpressed eRNA in HCC specimens. The pan-cancer assays revealed that SPRY4-AS1 was associated with glioblastoma multiforme (GBM), adrenocortical carcinoma (ACC), brain lower grade glioma (LGG) and mesothelioma(MESO). Positive associations were observed between SPRY4-AS1 and SPRY4 (its target gene) in 16 tumor types. Collectively, our findings reveal a novel eRNA SPRY4-AS1 for HCC progression and suggest that SPRY4-AS1 may be a potential biomarker and therapeutic target for HCC.

## Introduction

Hepatocellular carcinoma (HCC) is a high-prevalence cancer worldwide, especially in China (1, 2). Its incidence as well as mortality rate will still be on the increase over the forthcoming decades (3). Although more and more optimizations in the treatment strategies have resulted in distinct improvement of the survival of HCC patients, HCC remains a highly deadly neoplasm, with offensive malignancy and late diagnosis (4, 5). More optimized treatment strategies could be provided by prognosis prediction (6). In addition, these prognostic biomarkers may be potential targets for therapeutic intervention, thereby improving clinical outcomes (7). At present, as a commonly used biomarker, α-fetoprotein (AFP) displayed a relatively low sensitivity and specificity (8). Therefore, other valuable biomarkers must be investigated.

Long noncoding RNA (lncRNA) is in the molecule form with no possibility of being translated into a protein, and its nucleotides are over 200 (9). An example is enhancer RNA (eRNA) transcribed from putative enhancer regions (10). Its functions are still unclear, except that enhancer transcription is considered a noisy by-product of transcription machinery (11). Over the past years, substantial research has evidenced the crucial role of eRNAs in gene regulation for embryonic development and diseases (12, 13). Among thousands of eRNAs found in the cells of human beings, many display a mediating role in activating target genes (14). Specifically, eRNAs have been found to participate in the progression of cancers (15, 16). In recent years, the tumor-related functions of eRNAs have been reported in HCC. For instance, lncRNA SNHG17 was found to promote the proliferation and metastasis of HCC cells *via* regulating miR-3180-3p/RFX1 (17). LncRNA TTN-AS1 was observed to intensify sorafenib resistance in HCC through the modulation of miR-16-5p/cyclin E1 axis. However, what potential functions eRNA have in HCC remained unclear even though it is key to gene transcription control.

This research intended to identify prognostic eRNAs involved in HCC progression. Our findings suggest a strategy for targeting eRNA as a potential biomarker for the treatment of HCC patients.

## Materials and Methods

### Identification of Functional eRNAs in Hepatocellular Carcinoma Using Bioinformatics Technology

The Cancer Genome Atlas (TCGA) database (https://portal.gdc.cancer.gov/) was used to collect the data of 33 cancer types, including clinical and survival information, as well as RNA expression profiles. Here, 33 cancer types were included: pancreatic adenocarcinoma (PAAD), prostate adenocarcinoma (PRAD), rectum adenocarcinoma (READ), skin cutaneous melanoma (SKCM), stomach adenocarcinoma (STAD), colon adenocarcinoma (COAD), lymphoid neoplasm diffuse large B-cell lymphoma (DLBC), oesophageal carcinoma (ESCA), glioblastoma multiforme (GBM), bladder urothelial carcinoma (BLCA), head and neck squamous cell carcinoma (HNSC), kidney chromophobe (KICH), kidney renal clear cell carcinoma (KIRC), kidney renal papillary cell carcinoma (KIRP), brain lower grade glioma (LGG), liver hepatocellular carcinoma (LIHC), and uterine carcinosarcoma (UCS). The relevant eRNA information was obtained from the eRNA source literatures. By the use of the limma R software package, the eRNA expressing matrix and HCC patients’ survival information were combined. Kaplan–Meier assays were applied to determine the survival-associated eRNAs. Based on the median expression of each eRNA, patients were divided into high or low groups, low expression and high expression. By the use of Spearman’s correlation assays, candidate key eRNAs that were related to survival and target genes in HCC were obtained.

### Gene Enrichment Analysis

For exploring mechanisms whereby eRNAs may influence the clinical outcome of HCC patients, we used the “cluterprofile” R package to perform functional enrichment analyses of Kyoto Encyclopedia of Genes and Genomes (KEGG) and Gene Ontology (GO) (18, 19). The “ggplot2” R package was applied to identify the significant pathways. To avert a high false discovery rate (FDR) because of complex comparisons, we examined q-values. FDR-adjusted q-value <0.25 and p-value <0.05 were considered statistically significant.

### Tumor Samples

Ten paired HCC and matched normal non-tumor specimens were collected from 10 HCC patients (ages between 27 and 78 years old) at the Jinshan Hospital, Fudan University for RT-qPCR assays between June 2020 and May 2021. None of the patients received preoperative chemotherapy or radiotherapy. All cases were histopathologically confirmed as HCC by two independent pathologists. All tissues were preserved at -80°C until use. The Clinical Research & Ethics Committee of our hospital provided its approval of the present research. All patients offered informed consent in a written form.

### Quantitative Real-Time PCR Analysis

From all tumor and normal specimens, TRIzol reagent (Invitrogen, Suzhou, Jiangsu, China) was used to extract total RNA under the manufacturer’s instructions. RNA was reverse transcribed to cDNA from 2 μg of total RNA by Reverse Transcription Kits (Takara, Hangzhou, Zhejiang, China). qRT-PCR assays were carried out by a protocol from Power SYBR Green (Takara, Hangzhou, Zhejiang, China). The calculation and normalization of the relative expressions of SPRY4 antisense RNA 1 (SPRY4-AS1) and SPRY4 were achieved with 2^−ΔΔCt^ methods relative to glyceraldehyde 3-phosphate dehydrogenase (GAPDH). Specific primer sequences are shown in **Table 1**.

**Table 1:** Primers designed for qRT-PCR.

Name	Bidirectional primer sequence (5′–3′)
SPRY4: Forward primer	TCTGACCAACGGCTCTTAGAC
SPRY4: Reverse primer	GTGCCATAGTTGACCAGAGTC
SPRY4-AS1: Forward primer	GACCTGCTCGACCTGACCCTC
SPRY4-AS1: Reverse primer	CCACCTCGAACCACAATTCA
GAPDH: Forward primer	GGAGCGAGATCCCTCCAAAAT
GAPDH: Reverse primer	GGCTGTTGTCATACTTCTCATGG

GAPDH, glyceraldehyde 3-phosphate dehydrogenase; SPRY4-AS1, SPRY4 antisense RNA 1.

### Immunohistochemical Staining

Immunohistochemical staining was performed according to a previous study (20). Briefly, the cancer samples were fixed, paraffin-embedded, and sectioned. To obtain antigen retrieval, the sections were then incubated for 30 min. Then, for blocking endogenous peroxidase activities, we cultured the specimens 3% H_2_O_2_ for 20 min. Subsequently, primary antibodies targeting SPRY4 were applied to cultivate the sections of tumor specimens. Finally, after phosphate buffered saline (PBS) was applied to wash the sections, they are incubated with horseradish peroxidase (HRP)-conjugated secondary antibody followed by a light microscope for the photograph. Primary antibodies targeting SPRY4 were provided by Abcam (Cambridge, UK).

### SPRY4-AS1 Expression and Its Prognostic Value in Pan-Cancer

Firstly, the R limma package was used to obtain the expression data of SPRY4-AS1 and the corresponding target gene SPRY4 in pan-cancer, followed by combination with the survival data of pan-cancer. Samples were divided, based on the median value of SPRY4-AS1 expression, into two groups, high expression and low expression, followed by Kaplan–Meier methods determining the survival difference. The associations between SPRY4-AS1 and SPRY in pan-cancer were examined by the use of Spearman’s coefficient.

### Statistical Analysis

Through the SPSS statistical software package (standard version 18.0, SPSS Inc., Chicago, IL, USA) or RStudio (v.3.6.1, RStudio Team, 2016; RStudio Integrated Development for R; RStudio, Inc., Boston, USA), we conducted statistical analyses. Student’s t-test or chi-square test was applied to the analysis of group differences using SPSS. Combining Kaplan–Meier curves with log-rank tests with the “survival” R package, we analyzed survival results. Pearson correlation was used to analyze genes co-expressed with eRNAs in various tumors using RStudio. A p < 0.05 denoted statistical significance.

## Results

### Screening the Key eRNA in Hepatocellular Carcinoma

**Figure 1:**
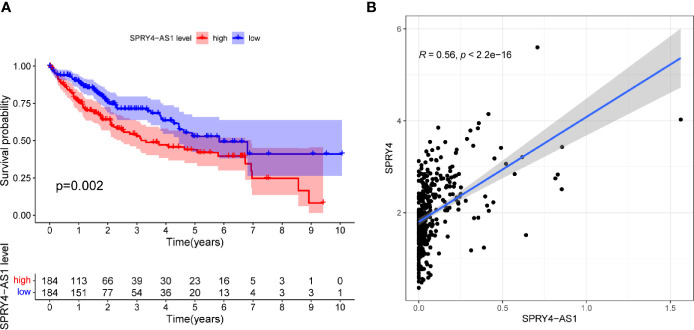
**(A)** Kaplan–Meier survival analysis of hepatocellular carcinoma (HCC) patients’ overall survival based on SPRY4 antisense RNA 1 (SPRY4-AS1) expression in The Cancer Genome Atlas (TCGA) datasets. **(B)** The correlation between SPRY4-AS1 and SPRY4 expression analyzed in TGCA datasets.

**Figure 2:**
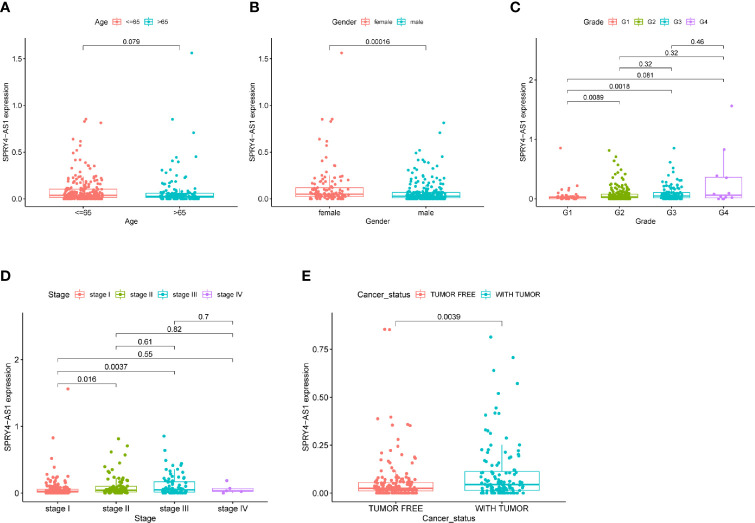
The associations between SPRY4 antisense RNA 1 (SPRY4-AS1) and clinical features. **(A)** Age. **(B)** Gender. **(C)** Grade. **(D)** Stage. **(E)** Cancer status.

To screen prognostic eRNAs, we analyzed TCGA datasets including 371 HCC cases (**Table 2**) and performed Kaplan–Meier methods, identifying 124 prognosis-related eRNAs with p < 0.05 (**Table S1**). To further identify 124 eRNAs with a distinct association with their target genes related to HCC, we performed Spearman’s correlation. SPRY4-AS1 exhibited the highest cor value. The results of Kaplan–Meier methods for SPRY4-AS1 suggested that the high-expression group had a shorter overall survival compared with the other group (p = 0.0002; **Figure 1A**). Besides, SPRY4 was found in **Figure 1B** to positively correlate with SPRY4-AS1 (R = 0.56, p < 2.2e10-15). Our group further examined the associations between HCC patients’ clinical features and the SPRY4-AS1 expression. We observed SPRY4-AS1 expression was not associated with age (p = 0.079; **Figure 2A**), However, we observed that the expression of SPRY4-AS1 in HCC was distinctly linked to gender (p = 0.00016; **Figure 2B**), grade (**Figure 2C**), stage (**Figure 2D**), and cancer status (p = 0.0039; **Figure 2E**).

**Table 2:** Baseline data of all HCC patients.

Characteristic		n	Proportion (%)
Total		371	100
Median follow-up (days)	557 (1–3,675)	371	100
Age	59.5 ± 13.0	371	100
Sex	Male	251	67.7
	Female	120	32.3
Tumor grade	I	54	14.6
	II	178	48.0
	III	122	32.9
	IV	12	3.2
	Unknown	5	1.3
Stage	I	174	46.9
	II	86	23.2
	III	84	22.6
	IV	5	1.3
	Unknown	22	5.9
T stage	I	183	49.3
	II	94	25.3
	III	80	21.6
	IV	12	3.2
	Unknown	2	0.5
N stage	Without metastasis	254	68.5
	With metastasis	4	1.1
	Unknown	113	30.5
M stage	Without metastasis	268	72.2
	With metastasis	4	1.1
	Unknown	99	26.7

HCC, hepatocellular carcinoma.

### Gene Enrichment Analysis

**Figure 3:**
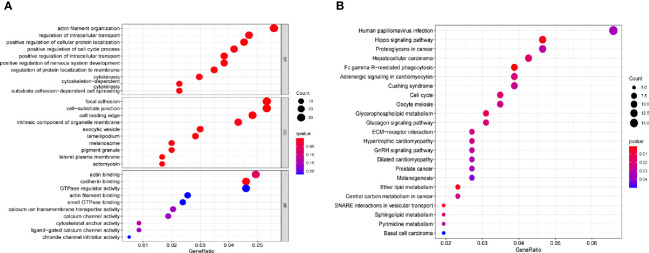
**(A)** Gene Ontology (GO) enrichment analysis. **(B)** The top 23 enriched Kyoto Encyclopedia of Genes and Genomes (KEGG) pathways.

A distinct association was found between 672 transcripts and SPRY4-AS1 (p < 0.05), including SPRY4. For the 1,407 target genes, KEGG pathway analysis and GO enrichment analysis revealed possible mechanisms responsible for the SPRY4-AS1 function. In biological process (BP), the association of terms with actin filament organization and the regulation of intracellular transport, cellular protein localization, cell cycle process, and intracellular transport was found; in Cellular Component (CC), they were related to focal adhesion, cell−substrate junction, cell leading edge, intrinsic component of organelle membrane, and exocytic vesicle. In Molecular Function (MF), term enrichment mainly involved actin binding, cadherin binding, GTPase regulator activity, and actin filament binding (**Figure 3A**). **Figure 3B** presents the 30 most important pathways. As suggested by KEGG assays, the BP mainly included Human papillomavirus infection, Hippo signaling pathway, and Proteoglycans in cancer (adjusted p < 0.001).

### The Distinct Upregulation of SPRY4-AS1 and SPRY4 in Hepatocellular Carcinoma Specimens

**Figure 4:**
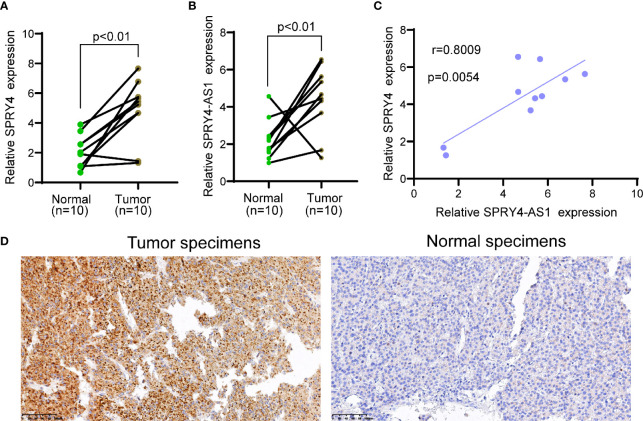
The upregulation of SPRY4 antisense RNA 1 (SPRY4-AS1) and SPRY4 in hepatocellular carcinoma (HCC) specimens. **(A, B)** RT-PCR for the levels of **(A)** SPRY4 and **(B)** SPRY4-AS1 in 10 pairs of HCC specimens and matched non-tumor specimens. **(C)** Association between SPRY4-AS1 and SPRY4 expression in 10 HCC specimens. **(D)** Immunohistochemical staining of 10 pairs of specimens.

To determine the expression of SPRY4-AS1 and SPRY4 in HCC specimens, we applied 10 HCC specimens and 10 non-tumor specimens to RT-PCR. As shown in **Figures 4A, B**, we observed that compared to the non-tumor specimens, the expression of SPRY4-AS1 and SPRY4 was distinctly increased in HCC specimens. Correlation assays revealed that SPRY4-AS1 expressions were positively associated with SPRY4 in 10 HCC tissues (**Figure 4C**). Furthermore, immunohistochemistry showed that the expressions of SPRY4 at the protein level were lower in non-tumor specimens than those in tumor specimens (**Figure 4D**).

### Pan-Cancer Verification

**Figure 5:**
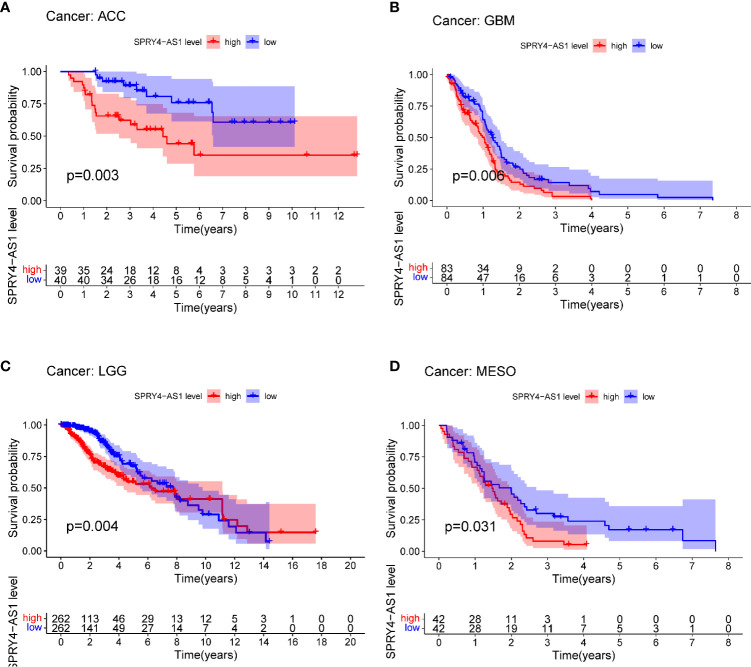
Survival curves for SPRY4 antisense RNA 1 (SPRY4-AS1) in pan-cancer, including ACC **(A)**, GBM **(B)**, LGG **(C)**, MESO **(D)**.

**Figure 6:**
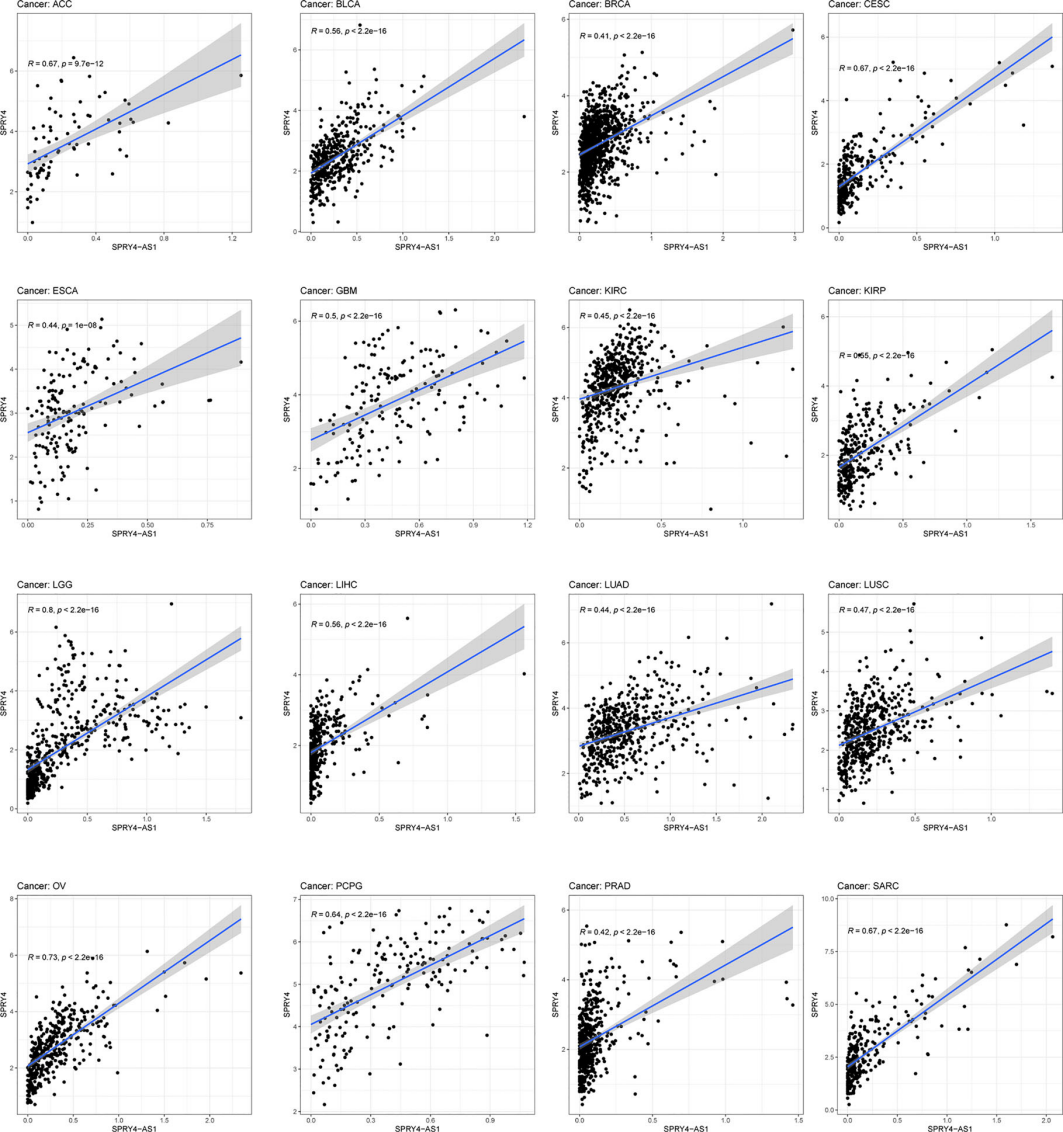
The association between SPRY4 antisense RNA 1 (SPRY4-AS1) and SPRY4 in pan-cancer.

The purpose of survival and correlation analyses was to demonstrate SPRY4-AS1’s prognostic value in pan-cancer. SPRY4-AS1 was found to associate with survival in ACC (**Figure 5A**), GBM (**Figure 5B**), LGG (**Figure 5C**), and MESO (**Figure 5D**). Besides, we observed that SPRY4-AS1 and SPRY4 were associated with 16 types of tumors (**Figure 6**).

## Discussion

HCC is a widely known malignant tumor in the world, and its incidence in many countries shows an upward trend (21). In the early stages, many HCC patients could achieve a favorable long-term survival after operative treatments and adjuvant therapies (22, 23). However, it is hard for patients with metastasis to receive operation, and their clinical outcomes remained very poor (24). Over the past decades, as the understanding of molecular mechanisms involved in HCC progression is deepened, various targeted therapies for HCC patients were developed (25, 26). Sensitive biomarkers are necessary to guide the application of targeted therapies. However, the related biomarkers were limited in clinical practice. In addition, the early detection also contributed to a favorable prognosis for HCC patients. Over the past 10 years, more and more studies focused on the huge potential of ncRNAs used as novel biomarkers for HCC patients (27, 28).

A growing number of studies have reported the involvement of eRNAs in several tumors. For instance, FOXP4-AS1, a prognosis-related eRNA, was reported to be lowly expressed in ovarian cancer specimens and predicted a shorter 5-year overall survival of ovarian cancer patients (29). The eRNA SMAD7e was found to promote the proliferation and metastasis of bladder cancer (30). Located in the tissue-specific enhancer of a tumor suppressor gene EMX2, eRNA EMX2OS was lowly expressed in kidney renal clear cell carcinoma specimens and associated with poor prognosis of tumor patients (31). However, in HCC, the effects of eRNAs were rarely reported.

In this study, we analyzed TCGA datasets and identified a novel HCC-related prognostic eRNA SPRY4-AS1. To date, the effects of SPRY4-AS1 in tumors have not been investigated. Moreover, SPRY4-AS1 was found to be the most critical eRNA candidate sequence in HCC, which regulated SPRY4. The expression of SPRY4-AS1 was found in clinical correlation analysis to vary with cancer status, gender, stage, and grade. Interestingly, we observed that the expression of SPRY4-AS1 in female patients was distinctly higher than that in male patients. The reason was unclear. More studies are needed to explore its reason. KEGG pathway enrichment results concluded the possible role of SPRY4-AS1 in the survival outcome of HCC patients. This could be achieved by Proteoglycans in cancer and Hippo signaling pathway. Previously, dysregulation of the Hippo pathway has been recognized in a variety of human cancers, including HCC (32, 33). The activity of Hippo signaling pathways was confirmed to modulate the proliferation and metastasis of HCC cells (34, 35). Furthermore, SPRY4-AS1 expressions in surgical specimens were verified by RT-PCR and immunohistochemistry, revealing an association with survival in ACC, GBM, LGG, and MESO. The above findings suggested that SPRY4-AS1 may be a novel prognostic biomarker for HCC patients.

Importantly, because this predictive model was based on the data from TCGA set, it remained unknown whether SPRY4-AS1 had similar predictive power beyond molecular subtypes in HCC patients of other Chinese hospitals. In addition, we did not unveil the molecular pathway through which SPRY4-AS1 exerted its function; this needs a further in-depth investigation.

## Conclusion

To conclude, a novel prognostic eRNA signature for HCC was examined in this paper. The research results may promote the prognostic evaluation of HCC and guide further study on targeted therapies in HCC.

## Data Availability Statement

The datasets presented in this study can be found in online repositories. The names of the repository/repositories and accession number(s) can be found in the article/**Supplementary Material**.

## Ethics Statement

The studies involving human participants were reviewed and approved by Jinshan Hospital, Fudan University. The patients/participants provided their written informed consent to participate in this study.

## Author Contributions

MY, J-BQ, and P-LS contributed to the conception. MY and SW contributed to the design and revision of the article. MY and J-BQ contributed to the analysis and interpretation of data. All authors contributed to the article and approved the submitted version.

## Funding

This work was supported in part by the Shanghai Municipal Health Commission (201740041).

## Conflict of Interest

The authors declare that the research was conducted in the absence of any commercial or financial relationships that could be construed as a potential conflict of interest.

## Publisher’s Note

All claims expressed in this article are solely those of the authors and do not necessarily represent those of their affiliated organizations, or those of the publisher, the editors and the reviewers. Any product that may be evaluated in this article, or claim that may be made by its manufacturer, is not guaranteed or endorsed by the publisher.

## Abbreviations

ACC, Adrenocortical Carcinoma; BLCA, Bladder Urothelial Carcinoma; BRCA, Breast Invasive Carcinoma; CESC, Cervical Squamous Cell Carcinoma and Endocervical Adenocarcinoma; CHOL, Cholangiocarcinoma; COAD, Colon Adenocarcinoma; DLBC, Lymphoid Neoplasm Diffuse Large B-cell Lymphoma; ESCA, Oesophageal Carcinoma; GBM, Glioblastoma Multiforme; HNSC, Head and Neck Squamous Cell Carcinoma; KICH, Kidney Chromophobe; KIRC, Kidney Renal Clear Cell Carcinoma; KIRP, Kidney Renal Papillary Cell Carcinoma; LAML, Acute Myeloid Leukaemia; LGG, Brain Lower Grade Glioma; LIHC, Liver Hepatocellular Carcinoma; LUAD, Lung Adenocarcinoma; LUSC, Lung Squamous Cell Carcinoma; MESO, Mesothelioma; OV, Ovarian Serous Cystadenocarcinoma; PAAD, Pancreatic Adenocarcinoma; PCPG, Pheochromocytoma and Paraganglioma; PRAD, Prostate Adenocarcinoma; READ, Rectum Adenocarcinoma; SARC, Sarcoma; SKCM, Skin Cutaneous Melanoma; STAD, Stomach Adenocarcinoma; TGCT, Testicular Germ Cell Tumours; THCA, Thyroid Carcinoma; THYM, Thymoma; UCEC, Uterine Corpus Endometrial Carcinoma; UCS, Uterine Carcinosarcoma; UVM, Uveal Melanoma.
