# Supplementary material for: SPRY4-AS1, A Novel Enhancer RNA, Is a Potential Novel Prognostic Biomarker and Therapeutic Target for Hepatocellular Carcinoma
Source: Front Oncol. 2021 Oct 4;11:765484. doi: 10.3389/fonc.2021.765484 (PMC8521147; doi:10.3389/fonc.2021.765484)
Supplement: Supplementary file 1 [file Table_1.docx]

Table S1. List of 124 survival-related eRNAs in HCC.

| gene | KM |
| --- | --- |
| EVX1 | 0.029356 |
| LINC01594 | 0.03552 |
| AL355574.1 | 0.003168 |
| ILDR2 | 0.014307 |
| AC114489.2 | 0.000212 |
| LINC02614 | 0.03821 |
| MTLN | 0.042389 |
| AL445524.1 | 0.009168 |
| MIR583HG | 0.016075 |
| AC007128.2 | 0.006043 |
| SNHG17 | 0.032923 |
| AL606468.1 | 0.026913 |
| AC114956.1 | 0.024866 |
| LGALSL-DT | 0.044701 |
| AC068985.2 | 0.015639 |
| MIR4435-2HG | 0.004299 |
| GRAMD1B | 0.014993 |
| LINC01090 | 0.008525 |
| STEAP1B | 0.000241 |
| LINC02037 | 0.005641 |
| SLC16A1-AS1 | 0.005093 |
| SLC25A24P1 | 0.001733 |
| LINC01010 | 0.002147 |
| AC096637.2 | 4.29E-09 |
| LINC02487 | 0.014308 |
| C12orf75 | 0.0006 |
| AL450322.2 | 0.005544 |
| AP003469.2 | 0.004378 |
| AL606807.1 | 0.025081 |
| LHFPL3-AS2 | 0.023274 |
| CRNDE | 0.038061 |
| AC006357.1 | 0.001288 |
| LINC01795 | 0.000142 |
| LINC02708 | 0.028125 |
| OSMR-AS1 | 0.010579 |
| LINC02810 | 0.008204 |
| LINC01152 | 0.048292 |
| AL139383.1 | 0.001577 |
| AL390879.1 | 0.019048 |
| AC004923.4 | 0.036107 |
| AC011352.3 | 0.009484 |
| LINC00958 | 0.017747 |
| AC122719.1 | 0.001854 |
| AC091806.1 | 0.029405 |
| AL118511.1 | 0.003206 |
| LINC01293 | 0.019998 |
| SPRY4-AS1 | 0.001744 |
| AL109933.1 | 0.012312 |
| FOXO3B | 0.028462 |
| AC004704.1 | 0.001822 |
| RRP7BP | 0.017068 |
| LINC02577 | 0.006131 |
| ELFN1 | 0.011812 |
| AL390198.1 | 0.013781 |
| AC145285.2 | 0.028454 |
| AC105345.1 | 0.000396 |
| MSN | 0.012063 |
| CDKN2B-AS1 | 0.00746 |
| LINC00200 | 0.009727 |
| LINC02768 | 0.03921 |
| AC004540.1 | 0.0072 |
| EMG1 | 0.002301 |
| AL391840.1 | 0.01901 |
| HOXB-AS4 | 0.009691 |
| AC005532.1 | 0.03552 |
| AC079760.2 | 0.031038 |
| AF279873.3 | 0.024312 |
| AL021707.2 | 0.005819 |
| HAGLROS | 0.027344 |
| AC006065.4 | 0.034136 |
| AC113346.1 | 0.043826 |
| AP006285.1 | 0.018784 |
| CECR7 | 0.011596 |
| AC009961.1 | 0.036403 |
| AC007405.2 | 0.038892 |
| SELENOOLP | 0.000372 |
| AC006206.2 | 0.02869 |
| LINC00261 | 0.003069 |
| AC084346.1 | 0.035849 |
| AC008957.1 | 0.036553 |
| SIRLNT | 0.001108 |
| SLC38A3 | 0.046543 |
| LINC01301 | 0.012363 |
| NBPF1 | 0.008106 |
| LINC01134 | 8.06E-05 |
| DCP1A | 0.000312 |
| AL137803.1 | 0.030002 |
| LINC02499 | 0.0096 |
| MIR100HG | 0.003266 |
| AL157937.1 | 0.017388 |
| AL035045.1 | 0.007636 |
| LINC01137 | 0.011554 |
| AC092957.1 | 0.02691 |
| AP001781.1 | 0.01054 |
| AC093607.1 | 0.011503 |
| HAS2-AS1 | 0.043051 |
| AL590705.2 | 0.00128 |
| LINC02154 | 0.003825 |
| LINC01094 | 0.026238 |
| LINC00601 | 0.03158 |
| LINC00160 | 0.018343 |
| LINC00460 | 0.044387 |
| BX322234.2 | 0.000413 |
| NUTM2B-AS1 | 0.021929 |
| RYK | 0.005345 |
| LINC00671 | 0.025372 |
| LINC01184 | 0.039149 |
| RHPN1-AS1 | 4.08E-06 |
| FOXP4-AS1 | 0.045414 |
| AC010789.1 | 1.37E-05 |
| CDK2AP1 | 0.046949 |
| AC079209.1 | 0.017775 |
| SERHL | 0.000653 |
| TMEM92-AS1 | 0.027752 |
| LINC02657 | 0.032109 |
| LINC01985 | 0.000233 |
| AC008667.1 | 0.021275 |
| LINC01357 | 0.017087 |
| MIR3945HG | 0.02691 |
| LRRC3-DT | 0.027486 |
| SLC2A1-AS1 | 0.00032 |
| AC007402.1 | 1.64E-06 |
| LINC01649 | 0.023747 |
| AC007848.1 | 0.011674 |
